# Combined Effects of Block-Based Programming and Physical Computing on Primary Students' Computational Thinking Skills

**DOI:** 10.3389/fpsyg.2022.875382

**Published:** 2022-06-08

**Authors:** Oliver Kastner-Hauler, Karin Tengler, Barbara Sabitzer, Zsolt Lavicza

**Affiliations:** ^1^Department of Media Education, University of Education Lower Austria, Baden bei Wien, Austria; ^2^Linz School of Education – Department of STEM Didactics, Johannes Kepler University Linz, Linz, Austria

**Keywords:** microbit, physical computing, block-based programming, computational thinking, computer science, primary digital education, integrated learning environment, assessment

## Abstract

Basic Digital Education (BDE) is already planned to be integrated with the forthcoming curriculum for Austrian primary schools (6–10 years) as it was already implemented for lower secondary schools (10–14 years) in 2018. BDE includes the most essential and novel developments of Computational Thinking (CT), which are fundamentally responsible for nurturing students' problem-solving skills. Thus, evaluating teaching materials, scaffolding guidelines, and assessments is becoming increasingly important for the successful implementation of CT in Austrian classrooms. This study is a part of a longitudinal multi-cycle educational design research project aiming to explore how to foster CT and to raise the awareness, importance, and confidence of teachers and students in applying CT for everyday uses. Our paper focuses on a sub-study in which teaching units for grade 3 and 4 students (8–10 years) were designed by combining an Open Educational Resource (OER) textbook and Physical Computing with the micro:bit device. The designed learning environment consists of three units and was implemented in two classes over 3 weeks. The two classes were further split into two groups each, to ensure better support during implementation. The class teachers received upfront teacher training and conducted pre- and post-test assessments with the students. The resulting data was then analyzed to gain insights into the effects on CT skills of the young learners. Results showed that combining block-based programming and physical computing devices could become a promising approach to promote computational thinking skills in lower school grades. Furthermore, the observed direction of the designed units supports low-barrier access to increase the desired uses of CT in classrooms.

## 1. Introduction

In our current knowledge and information-based society, Computational Thinking (CT) is becoming increasingly important due to the ongoing and widespread digitization. This digitization is also entering education, not least due to pandemic measures and required distance learning. As Wing (2006) amongst other leading computer scientists envisioned, Computational Thinking should establish itself as the fourth cultural skill (Bollin and Micheuz, 2019) over the coming years—next to reading, writing, and arithmetic. This involves the development and application of problem-solving skills and thinking strategies “*... everyone, not just computer scientists, would be eager to learn and use.”* (Wing, 2006). Broadening the set of skills, abilities, and attitudes that are key factors today for the successful implementation of digitization in education embraces the twenty-first Century Skills (P21–Battelle for Kids, 2019). These twenty-first century skills combined with the 5E instructional model for inquiry-based learning (Bybee, 2009) laid a substantial ground for the proposed learning design and make the haptic work with the physical computing device more effective for young learners.

CT has been implemented in a new curriculum (BMBWF, 2018) for lower secondary (10–14 years) in Austria as part of Basic Digital Education (BDE) and is already planned to be integrated into the forthcoming curriculum for primary (6–10 years). For broad introduction in lower secondary, an Open Educational Resource (OER) textbook for CT with the micro:bit (Bachinger and Teufel, 2018a) was developed, where the first author contributed example tasks in this resource (Bachinger and Teufel, 2018b). At the time of writing, there is still a lack of proven teaching material and scaffolding guidelines to conduct CT in class, particularly for primary teachers. Well-designed and working instructional material is urgently sought to provide support and get teachers started with the new tasks, especially for including less technically trained teaching staff (Papadakis, 2021). This paper aims to highlight how closing this gap can be achieved by establishing an integrated learning environment for CT with physical computing. Based on previous research cycles (Kastner-Hauler et al., 2021) and the feedback collected, selected examples from the OER textbook with the micro:bit were realigned and specially targeted for primary students. Students and teachers will be able to develop confidence by implementing the designed learning environment. Everyday CT use that builds on assessed material can emerge playfully. In addition, this way of administration supports the fulfillment of CT in an integrative way rather than seeing it as a separate subject.

In our work, we have been designing and developing a research project using the micro:bit physical computing device to support, promote, and evaluate the adoption of CT with BDE in schools. It is a longitudinal, multi-cycle research project to promote CT and increase awareness, importance, and confidence in its everyday applications. The sub-project and the focus of this article combines block-based coding with Makecode (Microsoft, 2016) and physical computing with the micro:bit (Sentance et al., 2017) in primary schools. Following this approach and building on the twenty-first century skills, the different aspects of CT can be demystified (Shute et al., 2017) and translated for classroom uses. In the following, we will demonstrate how the integrated learning environment guides the students gradually from computing at the desktop computer to physical computing with the micro:bit. We will show how the building blocks of the theoretical background work together to form a “*holistic approach of STEAM education through CT”* (Pears et al., 2019) and how the learning outcomes can be evaluated concerning CT (Zapata-Cáceres et al., 2021). STEAM refers to the fields of Science, Technology, Engineering, Arts, and Mathematics and is reportedly perceived as associated with micro:bit activities (Gibson and Bradley, 2017). Results give the comfortable impression, that inquiry-based learning enables the connection of all theory parts and provides playful discovery to foster the development of CT and problem-solving skills.

## 2. Context and Theoretical Background

### 2.1. Computational Thinking

The importance of CT in today's education is underlined by its increasing appearance in new curricula even at lower school levels. In Austria, CT was first implemented in 2018 as a mandatory element of Basic Digital Education (BDE) with the new curriculum for lower secondary education. There, CT is highlighted and defined as a separate area, alongside the user skills for common programs to manage everyday office life. The foundation for this trend in education was laid in an article by Wing (2006), which describes CT as a fundamental skill-set necessary for everyone to succeed in twenty-first century society. Wing further advances that CT skills can be applied to any problem, not just in computer science (CS), as a way of thinking within the problem solution process. The idea and mental concept behind CT originated from Papert (1980), who had also started the programming language Logo to promote and develop those skills. There, Papert also illustrates his practice-oriented constructionism that emphasizes on the learner's active role throughout the entire learning process while creating socially reflectible artifacts. The learning theory of constructionism connects the topics of this research, i.e., block-based programming, physical computing with the micro:bit, STEAM education, inquiry-based learning, and CT. For this purpose, a specially designed learning environment was rolled out and researched.

#### 2.1.1. CT Frameworks

The development of a universally accepted CT definition is not yet complete. However, in recent years, a fairly consistent picture for defining CT has emerged, and CT is thus becoming increasingly demystified (Shute et al., 2017). Shute et al. (2017) found that the most common aspects of CT are abstraction, decomposition, algorithms, and debugging—adding iteration and generalization with their research. Slight variations of this definition including frameworks, practices, and related concepts are described in Barr and Stephenson (2011), Brennan and Resnick (2012), Grover and Pea (2013), and Selby and Woollard (2013). A thorough overview of what (Wing, 2006) started with her claim for CT skills development and how the different frameworks relate to each other can be found at Palts and Pedaste (2020, p. 118)—proposing an all encompassing model for CT development. Recent research by Li et al. (2020) examined CT definitions and conclude to see CT more as a mental model of thinking and approach for problem-solving than a sole computing skill—supporting our holistic approach and STEAM integration (Pears et al., 2019).

#### 2.1.2. CT Assessment

When it comes to the assessment of CT, several researchers propose a system of assessments (Brennan and Resnick, 2012; Grover et al., 2015; Román-González et al., 2019) rather than one all-encompassing tool. For a comprehensive assessment of CT, Román-González et al. (2017a,b) developed, validated, and complemented a set of assessments to target all aspects of CT and the six levels of Bloom's (revised) taxonomy (Krathwohl, 2002). One test of the set of assessment tests developed is the Computational Thinking test (CTt). It was developed for 10–16 year-old students and is primarily used to test the levels 1 (Remember) and 2 (Understand) in Bloom's (revised) taxonomy. Due to the available time for the presented interventions of three units within 3 weeks, the higher levels 3 (Apply), 4 (Analyze), 5 (Evaluate), and 6 (Create) of the taxonomy cannot be expected to be achieved within this relatively short amount of time. Nevertheless, with the Beginners Computational Thinking test (BCTt) from Zapata-Cáceres et al. (2020, 2021) an adapted version of the CTt exists that specifically targets 5–10 year-old students. This perfectly fits the scope of this research, which targets 8–10 year-old students in primary school.

The BCTt follows the three-dimensional (3D) framework by Brennan and Resnick (2012) that groups CT aspects into three computational (ct) dimensions of ct-concepts, ct-practices and ct-perspectives. Brennan and Resnick (2012) identified seven ct-concepts that are highly useful for block-based coding projects and that also transfer to other non-programming contexts: *sequences, loops, parallelism, events, conditionals, operators, and data*. Applying the BCTt, 25 questions are assessed that can be answered independently of a programming environment either on screen or with paper and pencil. The assessment tests fully on ct-concepts, partially on ct-practices, and ignores ct-perspectives (Zapata-Cáceres et al., 2021). The BCTt is considered a scientifically sound and validated test for assessing computational thinking of primary school students (Román-González et al., 2017b; Zapata-Cáceres et al., 2020) and is recommended to be used within a *system of assessments* (Román-González, 2015) at the lower levels of Bloom's (revised) taxonomy.

### 2.2. Block-Based Programming

With the extended use of the computer in schools, the need to develop an understanding of its functioning and programming continues to increase. Especially in the school context, a distinction between text-based and block-based programming languages is essential for targeting the appropriate age group successfully. The text-based programming environments are preferably used for upper school levels and favor those who understand English well or are willing to learn it. Since the vocabulary of available commands is derived from the English language, one must have the commands memorized to be able to type them. Only the first few letters need to be typed from memory if auto-completion is offered by the programming environment. With block-based environments, there is no need to have prior experiences with the necessary vocabulary. The programmer can visually select from a given palette of command blocks, usually grouped by function, and then colored differently. Commands that do not show up in the programming environment simply do not exist. The block-based variants of programming languages are favored for lower school levels because of their lower entry barrier to start coding. A few common steps under teacher guidance are sufficient to then independently match the appropriate blocks (Weintrop and Wilensky, 2017) like a puzzle to construct a working program playfully. Additionally, in most block-based environments, the language of the blocks can be switched to other languages than English. Scratch from the MIT Media Lab (Resnick et al., 2009), which is considered as the mother of all block-based programming languages, is available in over 50 languages (Scratch-Wiki DACH, 2021). This compares to Makecode (Microsoft, 2016) for the micro:bit, which is used in the project and is also available in over 30 languages. Therefore, the integration of all learners in the event regardless of language level (Dasgupta and Hill, 2017) and vocabulary proficiency in English is assured. Block-based programming, even in early childhood ages (5–7 years), is reported to develop problem-solving, planning, and thinking strategies to gain social, language, and cognitive skills (Papadakis, 2022).

### 2.3. Physical Computing

Physical computing connects a computing device to the environment equipping it with the prerequisites for sensing (O'Sullivan and Igoe, 2004) and communicating (Igoe, 2017) and involves learners with the design and realization of tangible real world products from one's imagination (Przybylla and Romeike, 2014). A single-board computer such as the micro:bit is given access to the physical world utilizing sensors and controllers. These elements can sit directly on the single-board computer or be connected externally and augmented with electronic circuitry. The prevailing idea is that the machine can also handle some kinds of sensorimotor perceptions, albeit a bit more limited than humans. By engaging with physical matter and the environment, a way of human-machine interaction is obtained that is particularly conducive to grasping CS and CT concepts. Moreover, when a learner loads a self-created program onto the micro:bit, CS concepts take shape (Rock-Paper-Scissors Game—Supplementary Figure 1) and even get a face with the micro:bit LED display. We mainly focus on haptic tinkering, while playfully exploring the world of computing—functionalities and interrelationships can be developed more quickly in this constructionist learning setting. Furthermore, physical computing in the context of STEAM (Schulz and Pinkwart, 2015) promotes deeper understanding through the active creation of one's own learning experiences.

### 2.4. Inquiry-Based Learning and 5E Instructional Model

The essential features of classroom inquiry (National Research Council, 2000) were implemented by operationalizing the 5E instructional model (Bybee, 2009) and build an integral part of the proposed inquiry-based learning (IBL) design. Based on the model, the 5E learning cycle (BSCS.org, 2021) consists of five phases beginning with (1) engagement, (2) exploration, (3) explanation, (4) elaboration, and (5) evaluation. The framework of the 5E model provides enough elasticity to use open-ended, guided, or direct questioning depending on the situation and the actual student's needs.

The learning materials offered were developed following a combined approach to stimulate interest in further investigation and playful tinkering during the thought process of problem-solving. The starting material contains only a subset of the available material in hard copy for a sample exercise using the micro:bit, but sufficient to understand the problem at hand and get started. Next, the Wiki website based on the textbook contains additional materials, but this must be uncovered by the learner during exploration as needed and is not presented at first sight. This behavior is accomplished through “spoiler” links to be clicked before new information becomes available and builds on previous research (Kastner-Hauler et al., 2021). In this way, an emphasis is placed on student-centered and self-directed learning pathways through the design of the material (Reitinger et al., 2016).

## 3. Methodology

The implementation of a newly designed integrated learning environment for CT with physical computing is investigated utilizing a pre- and post-test approach. Through further refining and adjusting of the material, a theory desirably emerges and will be investigated in follow-up research on a larger scale. For now, the following question is explored in this paper:

RQ: To what extent do learning and teaching with the designed learning units for the micro:bit and Physical Computing effect students' Computational Thinking skills?

### 3.1. The Study

#### 3.1.1. Participants

The participants in the study were selected from a primary school of a district capital. The sample consisted of 45 third and fourth-grade students, 19 female, and 26 male, aged 8–10. Students had previous experiences in using computers, and tablets with digital media. However, they had no experiences with the micro:bit and Makecode programming preceding the start of the study. At the end of the intervention, data from 41 students were available for analysis. All of these students completed both tests, the pre- and the post-test. Thus, 20 third-grade children, 7 girls (35%) and 13 boys (65%), as well as 21 fourth-grade children, 11 girls (52%) and 10 boys (48%), participated in both tests. To meet all legal and ethical research requirements, permission was obtained to conduct this study with the students. Confidentiality of the survey was also assured, and the identities of the participants were not recorded anywhere in the test. Pseudonymous unique codes were used to allow comparison of pre- and post-test at the stages of data processing.

#### 3.1.2. Research Design

This pre- and post-test study is part of a long-term educational design research study that is conducted in iterative cycles (McKenney and Reeves, 2013, 2018). The research has been carried out since 2019 in selected primary and secondary schools with students in the age range of 8–14 years. Previously conducted studies on broadening awareness and application of computational thinking with physical computing included scaffolding material for learning and teaching CT with an Open Educational Resource (OER) textbook (Bachinger and Teufel, 2018a) and in combination with the Flipped Classroom method (Kastner-Hauler, 2020; Kastner-Hauler et al., 2021). Previous cycles have included studies of CT task difficulty recognition and design (Kastner-Hauler et al., 2020) and evaluation of the OER textbook usage to promote CT (Bachinger et al., 2021). For this paper, we focus on a sub-study in which instructional units were designed for third and fourth-grade students (ages 8–10) using the micro:bit. Two classroom teachers conducted three units each, and each class was divided into two groups for 3 weeks. Classroom teachers received in-service teacher training to avoid direct involvement of the authors and administered the pre- and post-tests (Zapata-Cáceres et al., 2021) for assessment. The resulting material was analyzed for observable effects on young learners' CT skills interacting with the learning environment (Basu et al., 2020, 2021).

#### 3.1.3. Measurement Tools

The Beginners Computational Thinking test (BCTt) version 2 by Zapata-Cáceres et al. (2021) is used as the assessment tool for CT measurement (Zapata-Cáceres, 2021). Version 2 of the BCTt consists of 25 tasks and can be completed within a lesson unit. Each task provides four single-choice answers as possible solutions that must be selected. The assessment contains the following computational concepts (Table 1): sequences (No. 1–6), loops (No. 7–18), and conditionals (No. 19–25). A chick and its mother hen are the two main characters in the assessment tasks. The primary goal is to bring the chick to its mother (Supplementary Figure 2) by solving the task conditions of the maze (Zapata-Cáceres et al., 2020). The assessment test was conducted online at the beginning and end of the intervention in the computer lab with the students. Each student entered a unique identification code *via* the web browser to allow direct correlation of the tests before and after the intervention. Anonymity and confidentiality of the data were maintained by processing the data pseudonymously. During the assessment, before starting each new category of computational concepts (Table 1), the test was paused and an explanatory example (Supplementary Figure 3) was solved together with the students upfront, as recommended by the creators of the BCTt.

**Table 1:** Computational concepts in BCTt (Zapata-Cáceres et al., 2021).

**Items**	**Loops**		**Conditionals**		
	**1. ** **Sequences**	**2. ** **Simple loops**	**3. ** **Nested loops**	**4. ** **If-then** **conditionals**	**5. ** **If-then-else** **conditionals**	**6. ** **While** **conditionals**
1–6						
7–11						
12–18						
19–20						
21–22						
23–25						

For the data analysis of the pre- and post-tests, all answers of students who took both tests were qualified. In the analysis, sociodemographic data on age and gender, as well as the resulting data of the items, were used. A *post-hoc t*-test for paired samples was utilized to determine a possible increase in CT skills. The *t*-test is an appropriate instrument for comparing the dependent samples of the pre- and post-tests with parametric data. The individual items of the BCTt were binary coded, e.g., with numbers of 1 for a correct response and of 0 for an incorrect response. Data analysis was performed using descriptive statistics and *t*-tests with the SPSS 27 software package.

### 3.2. Resources

#### 3.2.1. BBC Micro:Bit

The physical computing hardware used in the project is the BBC micro:bit. In 2013, the British Broadcasting Corporation (BBC) revived their past initiative “BBC Micro” from the 1980s to bring coding into every home, and school in the UK. The new initiative *Make it Digital* (BBC, 2015) aims to develop core skills in the STEAM fields (Tyrén et al., 2018) by inspiring creativity in the digital world. As of September 2016, the micro:bit project was transferred to the non-profit Micro:bit Educational Foundation (2016) independent of the BBC. Since then, the foundation has been working internationally to further disseminate and support the single-board computer—introducing coding even in primary school. Research on the use of the micro:bit in primary school shows support for collaboration, gamification, and individual work to develop problem-solving and programming skills (Kalogiannakis et al., 2021). The here described project works with both versions of the micro:bit, including the second version released in 2020.

The micro:bit combines all the features usually associated with a smartphone, such as Bluetooth connectivity, compass, acceleration, brightness, and temperature. With the listed features, the micro:bit qualifies as the ideal vehicle to promote physical STEAM activities with inquiry-based learning to bring CT to schools at an affordable price. The efficacy of this endeavor is confirmed by the first-year impact study (BBC and Discovery Research, 2017), which showed an increased interest in CS as a future subject option for students. After using the micro:bit in school, that was given for free to every grade 7 student in the UK, 70% more female and 25% more of all students considered CS (and CT) as subject focus after physical experimenting and live coding it.

#### 3.2.2. Integrated Learning Environment

Three learning units (Table 2) that show immediate success and further motivate students were created for the intervention. The design of the units follows the 5E instructional model from Bybee (2009, 2014) for inquiry-based learning (IBL). Earlier research from the authors developed materials that were based on an OER textbook and an accompanying wiki website for lower secondary students (Kastner-Hauler et al., 2021). Building on previous cycles, materials for primary students were redesigned by reducing and rephrasing too verbatim explanations and shifting to pictorials more appropriate for younger students. Textbook, wiki, and embedded tutorials combine the approach to encourage interest in further investigation and playful exploration during the problem solution process. First, the printed book contains only a selected subset of the available material for a sample task using the micro:bit, but enough to understand the given problem and get started after initial tutorials. Second, the wiki website based on the textbook contains additional material for each step, but this initially hidden content needs to be actively revealed by the learner. Therefore, the wiki provides “spoiler” hyperlinks that must be clicked before new information is accessible to the learner. In complement, the haptic aspect of physical computing intertwined with the handling of the micro:bit device by using its sensors, buttons, and the display is shown for each unit in Table 2. This way of structuring and integrating the learning environment supports self-driven exploration, tinkering, and inquiry-based learning paths (Reitinger et al., 2016) whilst playful search for a solution to a problem task.

**Table 2:** Lesson plan design—integrated learning environment.

**Units**	**Unit 1**	**Unit 2**	**Unit 3**
Objectives	- First program with makecode - Getting familiar with the micro:bit - Connect the micro:bit to USB/power - Transfer program to micro:bit	- Event-driven programming, Loops - Animate screen - Clear screen - Reset micro:bit - Display text - Repeat Loop	- Conditional programming, variables, sensors - If-then-else - Using accelerometer, light sensor - Using sound (computer only, micro:bit v1)
Activities	- Tutorial “flashing heart” - Hands-on micro:bit: computer connection with USB-cable - Drag and drop .hex program file onto micro:bit (USB) - Draw/display smiley - Draw/display stick figures	- When button A pressed → display built-in figure - When button B pressed → display other figure - When button A+B pressed → clear screen - When program reset/started → display single dot or letter - Animate Display → loop two figures	- Tutorial “rock, paper, scissors” → when “shaked” - Tutorial “sunlight sensor” → LEDs - When button pressed → play melody - Compose own melody
Physical computing	- Giving shape to algorithms - Use self-drawn stick figures	- Interact with device using buttons - Animate Screen	- Make the device feel (Shake, Light) - Make the device sound
Computational concepts	- Sequences - Loops (simple) - Events (on-start)	- Loops (advanced) - Events (buttons)	- Conditionals - Events (shake) - Variables (Data)
Computational practices^*^	- Being incremental and iterative	- Testing and debugging	- Reusing and remixing
Computational perspectives^*^	- Expressing	- Connecting	- Questioning
Learning outcome	1 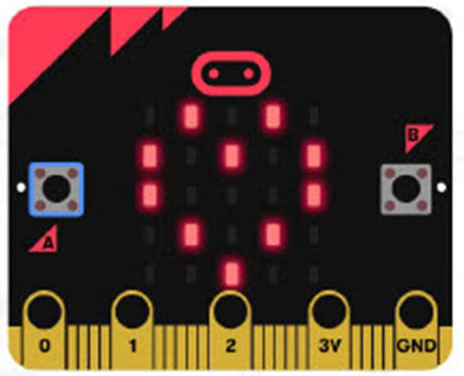	3 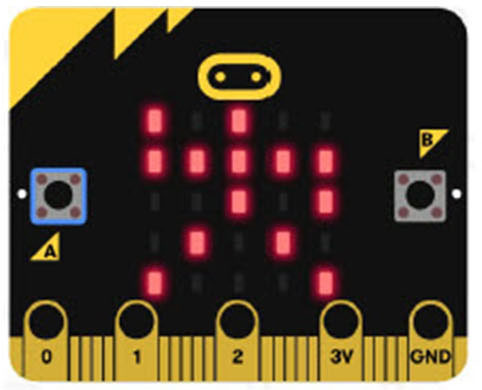	5 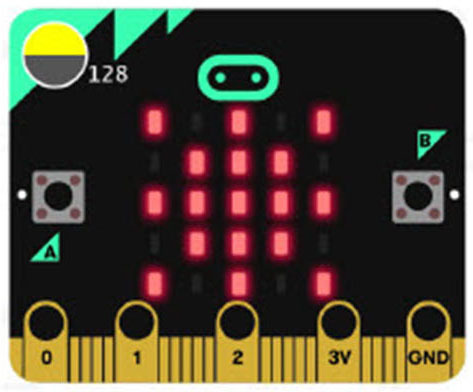
	2 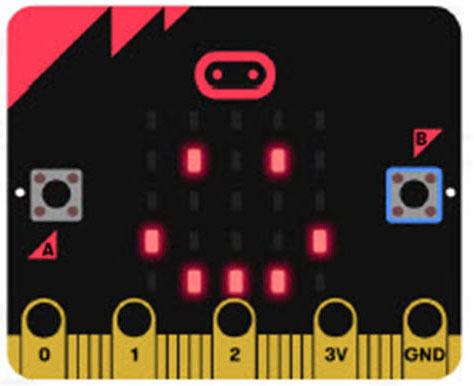	4 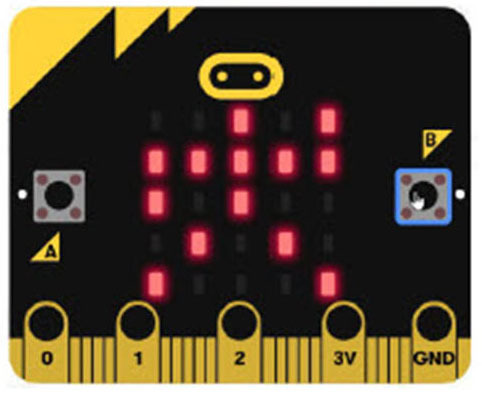	6 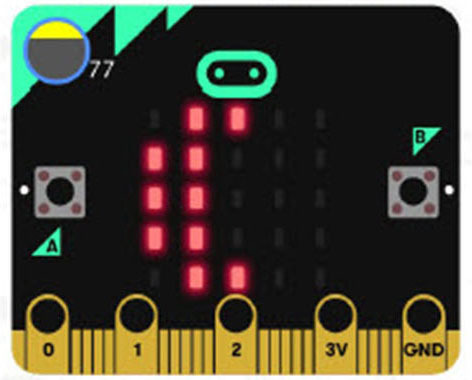

* *“Computational practices and perspectives” are trained throughout the entire intervention and are not explicitly matched to specific units*.

*For example, “Testing and debugging” can occur early or later on depending on the first result*.

*The same applies to “Connecting” and asking for help from seat neighbors, et cetera*.

*These CT aspects are interwoven with the inquiry-based constructionist learning process and are present across all units*.

**Figure 1:**
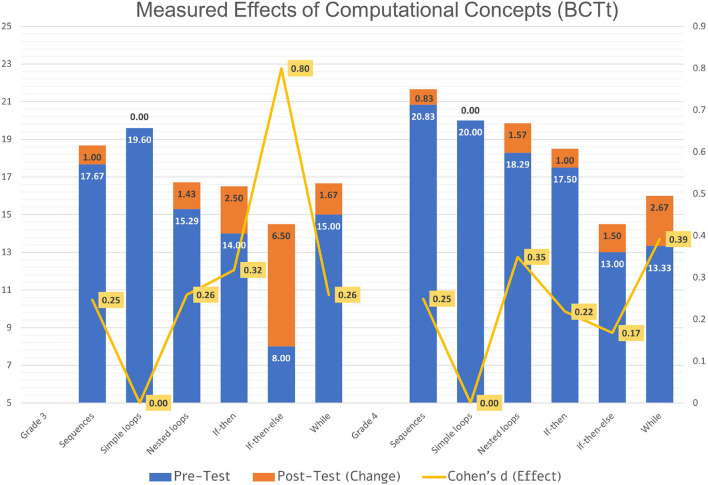
BCTt results—computational concepts progress.

*Figures 1 and 2: Heart – Grin: Sample artifact for unit 1 with block-based Makecode programming environment. Source: Micro:bit Educational Foundation, 2022, https://makecode.microbit.org/_7rLK17EcuXko. Reproduced with permission. Figures 3 and 4: Maxi1 – Max2: Sample artifact for unit 2 with block-based Makecode programming environment. Source: Micro:bit Educational Foundation, 2022, https://makecode.microbit.org/_a4tfis7zuVsj. Reproduced with permission. Figures 5 and 6: Moon – Sun: Sample artifact for unit 3 with block-based Makecode programming environment. Source: Micro:bit Educational Foundation, 2022, https://makecode.microbit.org/_D6agprWL4iWv. Reproduced with permission*.

#### 3.2.3. Lesson Plan Design Details

After an initial engagement and experimenting with how to connect the micro:bit and upload a program, unit 1 focused on display output with pre-set and custom 5 × 5-pixel graphics. Then, in unit 2, event-driven programming, loops, and animating the display output followed. In unit 3, advanced program constructs such as variables, and conditional branching were used and expanded to include physical computing with sensors. A period of 3 weeks was available for the overall intervention with three units (Table 2). The units are described in the following paragraphs in more detail.

Unit 1 consists of the tutorial “Flashing Heart.” When running through the tutorial, the individual steps are presented with short videos and are additionally supported by hiding all unnecessary command blocks. The hiding makes the initial orientation and the focus on the essential parts for the entry immensely easier. After that, the Makecode programming environment, including the micro:bit simulator which is available online, is explained in more detail. By plugging the micro:bit into the computer and uploading the program, the code can then be tested directly on the physical device and the pixel-drawing can be checked for refinement. Finally, the learners were guided to make their own drawing appear on the micro:bit's display.

Unit 2 introduces the concept of triggering an event by using buttons A and B, e.g., to start or clear the display. This is extended by using the reset button on the back of the micro:bit and by pressing both buttons A+B simultaneously. Finally, the display is animated, and two figures are shown alternately. The students are again encouraged to create their own figures after the joint exercise.

In unit 3, sensor functionality and more physical computing are used to start a program event-driven (through triggering of an event). For this purpose, the game “rock-paper-scissors” is translated to the micro:bit and the three symbols are displayed accordingly as soon as the micro:bit is shaken. A random number is generated, stored in a variable and the display is controlled with conditional branches. The next example uses the brightness sensor, which is hidden in some of the LEDs of the display and measures the brightness of the environment. If the brightness value falls below a certain threshold, the micro:bit should display a symbol for darkness, e.g., the moon, otherwise, the sun should appear. Finally, the micro:bit is used to output sound and create one's own melody. The sound output is most easily done on the computer with the micro:bit simulation since version 1 of the micro:bit used here does not have a speaker. At the end, the students were able to give feedback and describe what they liked best about programming the micro:bit with Makecode.

## 4. Results and Discussion

The BCTt was conducted twice in each group for both classes, before and after the three units of the intervention. The planned time for pre- and post-test was set equal to assure the same conditions for all students—although the post-test did not fully consume the planned time budget. This observable behavior is consistent with the findings of Zapata-Cáceres et al. (2020) who originally designed the assessment. Completing the intervention, pre- and post-tests were compared after initial data filtering for participation in both tests. The sample population appeared to be normally distributed with a sample size of *n* = 20 for third and *n* = 21 for fourth grade. Having met the prerequisites, it was possible to start the data analysis and examine the research question. Data processing was conducted from the first and second author under the four eyes principle. Findings from the elaborated data will be discussed subsequently.

### 4.1. Collected Data

The total score for each category of the BCTt shown in column *Avg. Sum* of the results in Table 3 has a maximum of 25 correct answers. The individual maximum was reached two times in the pre-test, only for the fourth grade, and six times in the post-test for both grades. The individual maximum score of 14 and 13 in the pre-test, and of 17 and 18 in the post-test occurred for the third grade and the fourth grade, respectively. A better post-test mean as a control source demonstrates the positive effect of the intervention, as does a higher average sum of correct answers in each category for both grade levels—except in the category of *Simple loops*. A possible explanation will be discussed later in the next section. Moreover, a higher post-test mean and a higher post-test average sum of all participants in the last row *Total* show the amount of positive effect more clearly.

**Table 3:** Evaluation of computational concepts—Grade 3 and 4.

**Computational** **concepts**	**Grade**	***N***	**BCTt**	**Mean**	**SD**	**Variance**	**Avg. sum**
Sequences	3	20	Pre	0.883	0.196	0.038	17.667
			Post	0.933	0.100	0.010	18.667
	4	21	Pre	0.968	0.085	0.007	20.333
			Post	0.992	0.036	0.001	20.833
Simple loops	3	20	Pre	0.980	0.062	0.004	19.600
			Post	0.980	0.062	0.004	19.600
	4	21	Pre	0.933	0.115	0.013	19.600
			Post	0.933	0.097	0.009	19.600
Nested loops	3	20	Pre	0.764	0.238	0.056	15.286
			Post	0.836	0.219	0.048	16.714
	4	21	Pre	0.864	0.199	0.040	18.143
			Post	0.932	0.116	0.014	19.571
If-then conditionals	3	20	Pre	0.700	0.299	0.089	14.000
			Post	0.825	0.294	0.086	16.500
	4	21	Pre	0.810	0.249	0.062	17.000
			Post	0.881	0.218	0.048	18.500
If-then-else conditionals	3	20	Pre	0.400	0.348	0.121	8.000
			Post	0.725	0.413	0.170	14.500
	4	21	Pre	0.619	0.415	0.173	13.000
			Post	0.690	0.402	0.162	14.500
While conditionals	3	20	Pre	0.750	0.284	0.080	15.000
			Post	0.833	0.229	0.053	16.667
	4	21	Pre	0.635	0.315	0.099	13.333
			Post	0.762	0.261	0.068	16.000
Total	3	20	Pre	20.000	2.991	8.947	400
			Post	21.950	3.120	9.734	439
	4	21	Pre	21.429	2.925	8.557	450
			Post	22.429	2.039	4.157	471

Further comparison of pre- and post-test with a *post-hoc* paired sample *t*-test (Table 4) showed the statistical significance of the intervention with *p* < 0.050. The intervention realized a total significance with a value of *p* = 0.006 for third grade and *p* = 0.029 for fourth grade. This clearly suggests that the designed units for Physical Computing with the micro:bit achieved a measurable positive effect on students' CT skills. Additional *t*-test comparison of the total number of correct answers shows a higher increase for third grade (*M* = 1.950, SD = 2.800) than for fourth grade (*M* = 1.000, SD = 1.949). Overall, third-graders were able to gain nearly double the amount of additional correct answers in the post-test than fourth-graders. On the other hand (Table 3), fourth-graders achieved a higher total number of correct answers upfront on the pre-test (*M* = 21.429, SD = 2.925) compared to the third-graders (*M* = 20.000, SD = 2.991).

**Table 4:** *Post-hoc* paired sample *t*-test (Grade 3 and 4).

**Computational** **concepts**	**Grade**	***N***	**BCTt**	**Mean**	**SD**	**Sig.** **(2-tailed)**	**Cohen's d**
Sequences	3	20	Pre + post	0.050	0.203	0.285	0.246
	4	21	Pre + post	0.024	0.096	0.267	0.249
Simple loops	3	20	Pre + post	0.000	0.092	1.000	0.000
	4	21	Pre + post	0.000	0.167	1.000	0.000
Nested loops	3	20	Pre + post	0.071	0.276	0.262	0.259
	4	21	Pre + post	0.068	0.195	0.125	0.349
If-then conditionals	3	20	Pre + post	0.125	0.393	0.171	0.318
	4	21	Pre + post	0.071	0.327	0.329	0.218
If-then-else conditionals	3	20	Pre + post	0.325	0.406	0.002	0.800
	4	21	Pre + post	0.071	0.427	0.452	0.167
While conditionals	3	20	Pre + post	0.083	0.322	0.262	0.259
	4	21	Pre + post	0.127	0.324	0.088	0.391
Total	3	20	Pre + post	1.950	2.800	0.006	0.696
	4	21	Pre + post	1.000	1.949	0.029	0.513

To obtain a deeper understanding of the different aspects of the BCTt, a detailed group comparison of the six computational concepts (Table 1) was performed. The paired sample *t*-test values for mean, standard deviation, and significance grouped by the six categories of computational concepts are presented in Table 4. Although the comparison of the total of all categories showed statistical significance in the overall result, almost all the single categories lack a significant increase except one category. The category *If-then-else* provides a significance value of *p* = 0.002 for third grade.

To better illustrate the measured outcome of the interventions' effect on students' Computational Thinking skills, here characterized by the BCTt's categories of computational concepts, a combined chart of pre-test, post-test, and Cohen's d effect was elaborated in Figure 1. This graphical presentation depicts the highest gain of competencies in the category *If-then-else* for third grade (Change = 6.50, i.e., from *M* = 8.00 to *M* = 14.5) and in the category *While* for fourth grade (Change = 2.67, i.e., from *M* = 13.33 to *M* = 14.00). All other categories for both grades show an increase and effect (Cohen's d)—except the category *Simple loops*. This category shows no explicit gain in competencies, but the connected sister-category of *Nested loops* shows an increase for both grades. Third-graders scored lower on pre- and post-test in almost all categories except for the category *While*, whereas fourth-graders scored lower than third-graders for both, pre- and post-test.

The overall effect of the intervention is measured with a Cohen's d value of 0.696 for third grade and 0.513 for fourth grade (Table 4). Having a Cohen's d value of >0.5 suggests a medium effect for both grades, achieving almost a large effect (Cohen's d > 0.8) for third grade. This outlines an overall increase in the categories of computational concepts related to the intervention, particularly the Computational Thinking skills that are effected by programming the micro:bit.

### 4.2. Discussion

This study aimed to evaluate newly designed teaching material for CT with the micro:bit and Physical Computing used in primary school. The BCTt was applied to assess the amount of the learning effect on students' CT skills with a 3 week intervention. The BCTt is a validated assessment (Zapata-Cáceres et al., 2020) for CT skills following the 3D framework focusing on different categories of computational concepts, partially on computational practices, and ignores computational perspectives (Zapata-Cáceres et al., 2021). Setup and implementation of the BCTt are easy to apply, making it widely available to researchers and teachers in the field. The above characteristics clearly signal the potential of the BCTt and its value for curriculum design and material development with CT assessment. In the following, the findings and limitations of the demonstrated study will be discussed.

#### 4.2.1. Findings

The study indicates an increase in CT skills for both grades comparing the total sum of the BCTt score for pre- and post-test (Table 3). The figures suggest a higher effect for third-graders compared to fourth-graders, albeit both effect scores are within medium range. Both classes had no prior experiences with the micro:bit and Makecode programming before this intervention, but the primary school under study is equipped with Bee-Bots programmable floor robots. Teachers from the afternoon care regarding fourth grade were reportedly curious about this cute little robot and showed interest in trainings on this subject (Tengler et al., 2021) after collecting additional feedback. Therefore, we assume that fourth-graders in this study had first contact with the Bee-Bot during unofficial afternoon hours. On the other hand, the measured higher effect for third grade can be explained through a possible ceiling effect of fourth-graders in the assessment, which was also reported by the designers of the BCTt during the evaluation of their assessment instrument (Zapata-Cáceres et al., 2021). In addition, fourth-graders have more or less a year of development advantage given their age. The authors suggest further investigation to clarify these findings in the outlook section.

The category of *Simple loops* has the same value for pre- and post-test for both grades. Representing the highest achieved amount for third grade and second-highest for fourth grade suggests a possible explanation for the unmeasured difference. As a very high score on the pre-test diminishes the available room left for improvement on the post-test. Moreover, the trained computational concepts with Makecode and the micro:bit were mainly located in the sister-category *Nested loops*. Or simply, the high amount already known in this category makes much more effort necessary to improve measurably what the 3 week intervention was not able to provide.

Another interesting finding is depicted in Figure 1 in the category *If-then-else*. The improvement for third grade in this category is extremely high yet it achieves overall the lowest score in pre-test. Having a large available range for improvement on the post-test is the nature of a relatively low pre-test score. In addition, the training deepens more on the computational concepts of the sister-categories *If-then* and *While* conditionals. The highest improvement for fourth grade can be seen in the category *While*, which suggests overall good effectiveness of the whole intervention. Since the difficulty level increases from one category to the next, the last category *While* can be seen as an indicative marker for the whole journey conveyed.

Problem-solving as a general cognitive ability is considerably linked to CT in the context of programming (Kalelioglu et al., 2016). It is important to train such problem-solving skills at an early stage of childhood to generate action-relevant knowledge and gain non-verbal intelligence and CT skills. Our presented study provides evidence that supports this point of view, which also compares to similar findings of other studies (e.g., Román-González et al., 2017b; Tsarava et al., 2019; Zapata-Cáceres et al., 2021).

#### 4.2.2. Limitations

Some findings and observations of the study could be further explored for better understanding and overcoming limitations. The study showed that third-graders profited more than fourth-graders from the training. Therefore, it would be interesting to extend research into age group appropriate task design and tailor more specific for the participants' grades as the inventors of the BCTt suggest to prevent ceiling effects. This applies to gender-specific tailoring as well, not touched in examination anywhere here. The available time for the intervention is another point to consider in more detail. The 3 weeks of training could be extended to six or even 3 months in further studies. And finally, the target group availability was limited due to pandemic constraints and the so resulting sample size of the participants should be enlarged in upcoming cycles of research.

The BCTt is only one part of the set of three complementary assessment tools as researched by Román-González et al. (2017a), but it is sufficient to trial the direction of newly created learning materials. Given the relatively short amount of available time for the intervention higher levels of Bloom's (revised) taxonomy cannot be expected to be achieved. Therefore, the BCTt is an appropriate instrument for assessing the reported intervention on the lower levels of Bloom's (revised) taxonomy linked to CT and problem-solving.

## 5. Conclusions and Outlook

The study presented in this article is part of a longitudinal multi-cycle educational design research (McKenney and Reeves, 2018) on teaching materials and scaffolding guidelines for CT implementation in the classroom. The overall research goal of the entire project aims to foster CT and to raise the awareness, importance, and confidence of teachers and students to apply CT as an everyday skill. The first step was to identify existing teaching materials and teacher readiness for CT uses in class. The need to vary the difficulty levels of existing example tasks from an identified source, the OER textbook and wiki for the micro:bit (Bachinger and Teufel, 2018b), was denoted and further investigated (Kastner-Hauler et al., 2020). The next step, during pandemic constraints, was to adapt an example task (Kastner-Hauler et al., 2021) for flipped classroom delivery (Lage et al., 2000; Bergmann and Sams, 2012; Buchner, 2018). In this paper, we focus on a sub-study in which three units for primary students (grades 3 and 4) were designed using an OER textbook for CT with the micro:bit—originally designed for lower secondary level (grades 5–8). The interventions laid out in Table 2 were conducted by emphasizing the haptic and sensing elements of Physical Computing with the micro:bit device. The assessment of CT took place before and after the interventions with the BCTt (Zapata-Cáceres et al., 2021). The consecutive steps, not included in this article, will be developed and assessed during in-service teacher training for CT with design guidelines iteratively derived throughout the entire project. In the next phase of our study, we will also investigate other CT assessments as mentioned and their operational applicability to further drive CT content development for classroom use.

With this study, we provide empirical evidence that teaching block-based coding combined with Physical Computing can foster CT skills of young learners of third and fourth grade (8–10 years) in primary school. Answering the research question, a significant value for the overall effect of the intervention was obtained and the increased values for the trained categories show a positive effect. A successful introduction to the concepts of CT and programming can be provided by the newly designed teaching material, even in upper primary school. The results of this study provide also the opportunity to further extend the research to lower secondary with a specifically targeted age group (10–14 years) assessment in upcoming cycles, i.e., the Computational Thinking test (CTt) (Román-González, 2015) or the abbreviated CTt (Tsarava et al., 2022). Further extending assessments to all six levels of Bloom's (revised) taxonomy could include the use of Bebras Tasks (Dagiene and Sentance, 2016; Bebras.org, 2022), Dr. Scratch (Moreno-León et al., 2015) or possibly Dr. Micro:bit—a version of Dr. Scratch to be developed in the future. Further cycles of materials development should incorporate self-authored tutorials within Makecode to eliminate clutter and focus on ct-concepts to learn. After that, *sidedoc* elements (collapsible, floating menus) within Makecode should enhance the use of the OER materials, eliminating the clutter and the need to switch between browser pages—reading instructions and programming can so be accomplished on one page.

Learning and teaching CT in the context of Physical Computing can be mastered in upper primary school levels (grades 3 and 4) with the here assessed materials. Results gave us positive feedback that our intervention had a substantial impact toward the desired direction. The combination of block-based programming with Makecode and the micro:bit are promising approaches to foster CT skills and introduce Basic Digital Education in primary schools with a playful, enjoyable, and tangible learning environment. Both students and teachers could be consequently empowered to experience a confident and good feeling in developing and applying their Computational Thinking skills whilst problem-solving.

## Data Availability Statement

The original contributions presented in the study are included in the article/Supplementary Material, further inquiries can be directed to the corresponding author/s.

## Ethics Statement

The studies involving human participants were reviewed and approved by University of Education Lower Austria. Written informed consent to participate in this study was provided by the participants' legal guardian/next of kin.

## Author Contributions

OK-H: conceptualization, methodology, writing and re-writing, visualization, and project administration. OK-H and KT: investigation. OK-H and ZL: editing. BS and ZL: supervision. All authors have read and agreed to the published version of the manuscript.

## Funding

Open access funding provided by Johannes Kepler University Linz, Austria.

## Conflict of Interest

The authors declare that the research was conducted in the absence of any commercial or financial relationships that could be construed as a potential conflict of interest.

## Publisher's Note

All claims expressed in this article are solely those of the authors and do not necessarily represent those of their affiliated organizations, or those of the publisher, the editors and the reviewers. Any product that may be evaluated in this article, or claim that may be made by its manufacturer, is not guaranteed or endorsed by the publisher.
