# Supplementary material for: Combined Effects of Block-Based Programming and Physical Computing on Primary Students' Computational Thinking Skills
Source: Front Psychol. 2022 Jun 8;13:875382. doi: 10.3389/fpsyg.2022.875382 (PMC9216230; doi:10.3389/fpsyg.2022.875382)
Supplement: Supplementary file 1 [file Data_Sheet_1.pdf]

Supplementary Material

1 SUPPLEMENTARY FIGURES

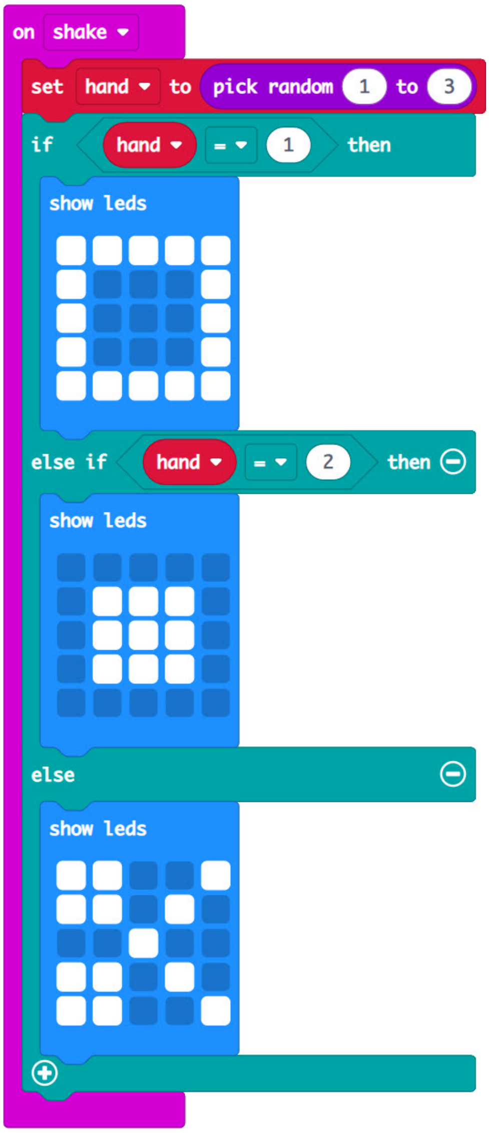

Figure S1. Rock-Paper-Scissors: Sample artifact for unit 3 with block-based Makecode programming environment. Source: Micro:bit Educational Foundation, 2022 , `https://makecode.microbit.org/_JR3MtvTrMFX4`. Reproduced with permission.

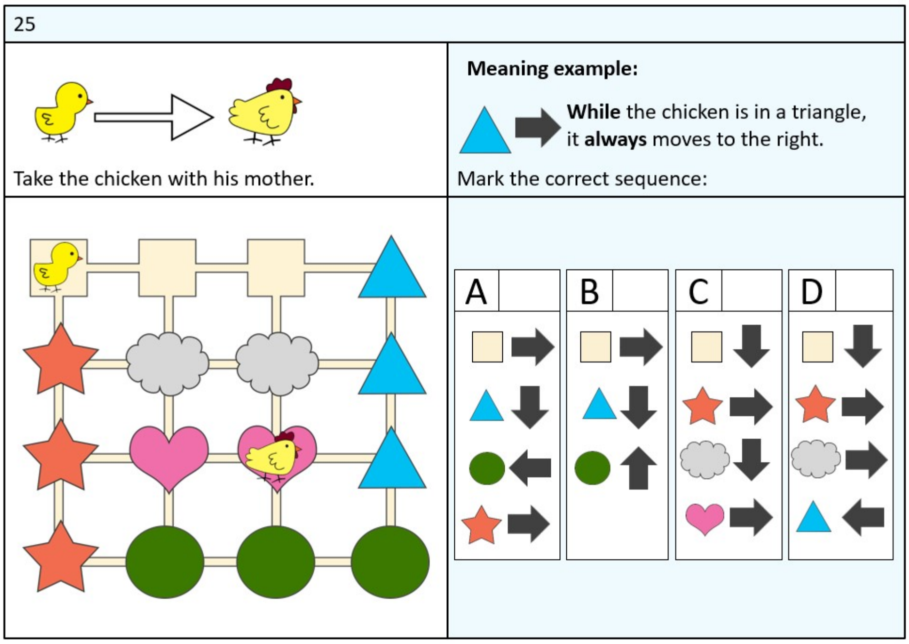

Figure S2. Sample question Q25 from BCTt assessing the program constructs *loops* (while) and *conditions* (always).
Source: Zapata-Cáceres, M., Martín-Barroso, E., and Román-González, M. (2020). "Computational thinking test for beginners: design and content validation," in 2020 IEEE Global Engineering Education Conference (EDUCON) (Porto), 1905–1914. Reproduced with permission.

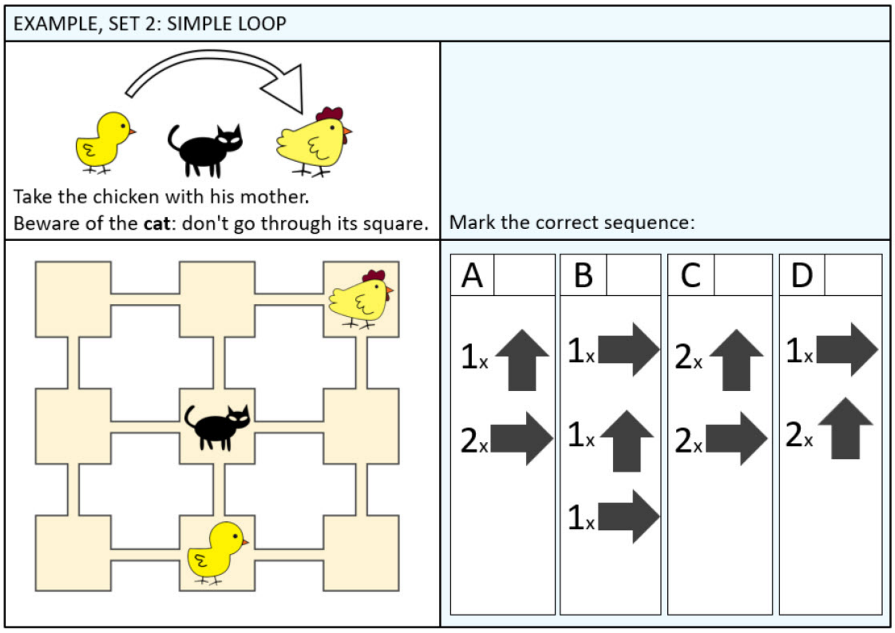

Figure S3. Sample explanation upfront assessment questions 7-11 from BCTt assessing the program constructs – *simple loops*.
Source: Zapata-Cáceres, M., Martín-Barroso, E., and Román-González, M. (2020). "Computational thinking test for beginners: design and content validation," in 2020 IEEE Global Engineering Education Conference (EDUCON) (Porto), 1905–1914. Reproduced with permission.
